# O069. Menstrual cycle affects cortical excitability differently in females with migraine and in healthy controls: a new perspective by cross modal sound induced flash illusions

**DOI:** 10.1186/1129-2377-16-S1-A141

**Published:** 2015-09-28

**Authors:** Simona Maccora, Carlo Mannina, Nadia Bolognini, Piera Paladino, Roberta Baschi, Giuseppe Cosentino, Brigida Fierro, Giuseppe Vallar, Filippo Brighina

**Affiliations:** Dipartimento di Biomedicine Sperimentali e Neuroscienze Cliniche (BioNeC), Università di Palermo, Palermo, Italy; Dipartimento di Psicologia, Università di Milano-Bicocca, Milan, Italy; Laboratorio di Neuropsicologia, IRCCS Istituto Auxologico Italiano, Milan, Italy

## Introduction

The sound-induced flash illusions (SIFI) represent a valid tool to explore multimodal perception and are critically dependent on visual and acoustic cortical excitability [1, 2]. In a previous study [3], we observed a significant reduction of illusions in migraine patients with respect to healthy controls, probably due to a condition of visual cortex hyperexcitability. Aim of the present study was to evaluate SIFI perceptions in healthy women and patients with menstrual migraine and to describe the effects of cyclical change of steroid hormones and cortical responsiveness.

## Materials and methods

Nineteen women (11 affected with menstrual migraine, 8 healthy controls) were enrolled. Serum determination for sexual hormones (estradiol, progesterone) and a SIFI trial were performed in all participants in two different sessions on the 14th and 27th day of menstrual cycle.

## Results

Healthy women showed more illusions in the premenstrual (27th day) than in the luteal phase (14° day) (p < 0.01). Migraine patients did not show any difference during the two phases of menstrual cycle; they saw significantly less fissions illusions (p < 0.001) with respect to healthy women at 27th, but not at 14th day of menstrual cycle.

## Conclusions

Results in healthy subjects are in line with hormonal effects on cortical excitability. During late follicular phase, the increase of estradiol could determine visual cortex hyperexcitability corresponding to a reduction of SIFI. Conversely, premenstrual fall of estradiol would account for restored illusions. Persistence of a reduced illusory susceptibility in both phases of menstrual cycle in migraine patients would underlie a reduced responsivity of visual cortex to hormonal fluctuation.

Written informed consent to publish was obtained from the patient(s).
